# Whole Genome Re-sequencing and Bulk Segregant Analysis Reveals Chromosomal Location for Papaya Ringspot Virus W Resistance in Squash

**DOI:** 10.3389/fpls.2022.848631

**Published:** 2022-05-19

**Authors:** Swati Shrestha, Yuqing Fu, Vincent Njung’e Michael, Geoffrey Meru

**Affiliations:** Horticultural Sciences Department and the Tropical Research and Education Center, University of Florida, Homestead, FL, United States

**Keywords:** *Cucurbita*, Nigerian Local, aphid-transmitted potyvirus, genetic loci, marker-assisted selection

## Abstract

Squash (*Cucurbita moschata*) is among the most important cucurbit crops grown worldwide. Plant pathogen, Papaya ringspot virus W (PRSV-W) causes significant yield loss in commercial squash production globally. The development of virus-resistant cultivars can complement integrated disease management and mitigate losses due to viral infections. However, the genetic loci and molecular markers linked to PRSV-W resistance that could facilitate marker-assisted selection (MAS) for accelerated cultivar development are unknown. In this study, quantitative trait loci (QTL), molecular markers, and candidate genes associated with PRSV-W resistance in squash were identified in an F_2_ population (*n* = 118) derived from a cross between Nigerian Local accession (resistant) and Butterbush cultivar (susceptible). Whole genome re-sequencing-based bulked segregant analysis (QTLseq method; *n* = 10 for each bulk) and non-parametric interval mapping were used to identify a major QTL associated with PRSV-W resistance on chromosome 9 (*QtlPRSV-C09*) (*p* < 0.05) of *C. moschata*. *QtlPRSV-C09* extended from 785,532 to 5,093,314 bp and harbored 12,245 SNPs among which 94 were high-effect variants. To validate *QtlPRSV-C09*, 13 SNP markers were assayed as Kompetitive allele-specific PCR (KASP) markers in the F_2_ population and tested for the association with PRSV-W resistance. Among these, two KASP markers (Ch09_2080834 and Ch09_5023865-1) showed significant association with PRSV-W resistance (*p* < 0.05). The two SNPs were located within exons of putative disease-resistant genes encoding the clathrin assembly family and actin cytoskeleton-regulatory complex proteins, which are implicated in disease resistance across plant species. The findings of this study will facilitate MAS for PRSV-W resistance in squash and allow further understanding of the functional mechanisms underlying potyvirus resistance in *Cucurbita* species.

## Introduction

Squash and pumpkin (*Cucurbita* species) constitute an economically important horticultural crop grown in most of the subtropical and temperate regions of the world (Kates, 2019). *Cucurbita moschata* is among the major cultivated species of squash valued for culinary, processing, and ornamental purposes worldwide (Whitaker and Robinson, 1986). Flowers and tender shoots of squash are relished as vegetables while fruits are consumed either baked or cooked. Fruits of squash are also used in pies, soups, and jams (Lim, 2012). The flesh and leaves of *C. moschata* are a good source of vitamins, amino acids, flavonoids, and carbohydrates, whereas the seeds have significant levels of unsaturated fatty acids and antioxidants (Stevenson et al., 2007; Yadav et al., 2010; Vinayashree and Vasu, 2021).

The US is among the top-five producers of squash in the world with 116,600 acres of land under production delivering an annual value of more than 411.8 million dollars in 2020 (USDA-National Agricultural Statistics Service [NASS], 2021). Disease outbreaks caused by potyviruses (Potyviridae family) are the major limiting factors for squash production in the US (Boyhan et al., 2009). More than 165 potyvirus species are known to infect plants (Nigam et al., 2019); however, the most devastating for Cucurbita crops are Papaya ringspot virus W (PRSV-W), Zucchini yellow mosaic virus (ZYMV), and Watermelon mosaic virus (WMV) (Paris, 2008). These three viruses can infect both vegetative and reproductive parts of the plants, which cause severe mottling in leaves, stunted growth, and deformed fruits, and can cause 100% yield loss when susceptible cultivars are grown (Seda-Martínez et al., 2021). Chemical and cultural management methods aim at suppressing aphid-vector populations but have limited efficacy due to the obligate intracellular nature of the viruses (Rubio et al., 2020). Utilizing genetic host resistance is the most reliable and cost-efficient way of minimizing losses due to the viruses (Brown et al., 2003; Collard et al., 2005; Pachner et al., 2011; Rubio et al., 2020).

Resistance to PRSV-W, ZYMV, and WMV has been reported in Nigerian Local, a landrace of *C. moschata* from Nigeria (Munger and Provvidenti, 1987; Brown et al., 2003). Resistance to PRSV-W in Nigerian Local is controlled by a single recessive gene (prv) with modifiers (McPhail-Medina et al., 2012; Seda-Martinez, 2019), whereas resistance to WMV is controlled by a single dominant gene (Wmv) (Behera et al., 2012). On the other hand, resistance to ZYMV in Nigerian Local is conferred by two genes designated Zym-0 and Zym-4, with the latter only conferring resistance in complementary interaction with a recessive gene designated zym-5 in ZYMV-susceptible cultivar, Waltham Butternut (Brown et al., 2003; Pachner et al., 2011). Traditional breeding strategies for developing cultivars of squash resistant to potyviruses are costly and dependent on resource-intensive phenotypic selection methods. For example, the first commercial squash cultivars integrating virus resistance from wild germplasm took nearly 20 years to develop using traditional breeding approaches (Brown et al., 2003). This is due to interspecific barriers across *Cucurbita* species and the non-allelic nature of resistance to the three potyviruses (Brown et al., 2003). However, molecular tagging of resistant genes through marker-assisted selection (MAS) can reduce the length of selection cycle by allowing the identification of desirable plants at the seedling stage or through seed-based genotyping (Collard et al., 2005; Meru et al., 2013; Martinez et al., 2021). Molecular markers tightly linked to ZYMV resistance in Nigerian Local have recently become available for MAS (Pachner et al., 2011; Shrestha et al., 2021). However, the quantitative trait loci (QTL) and molecular markers linked to PRSV-W and WMV resistance in Nigerian Local are currently unknown, thus limiting potential genetic gain through MAS.

The advances in next-generation sequencing technologies and availability of cost-effective genotyping platforms have facilitated the development of rapid genomic tools for crop advancement (Buermans and Den Dunnen, 2014). The recent availability of a reference genome for *C. moschata* (Sun et al., 2017) has accelerated the rapid development of MAS tools for economically important traits through linkage mapping (Sáez et al., 2020; Vogel et al., 2021) and QTLseq (Ramos et al., 2020; Shrestha et al., 2021). QTLseq is a genetic mapping method that integrates whole genome sequencing data into traditional bulk-segregant analysis to provide robust and rapid identification of genomic loci associated with the traits of interest (Takagi et al., 2013). Briefly, the method involves developing a mapping population segregating for a trait of interest and identifying individuals expressing the extreme phenotype. DNA from these individuals is bulked into high and low pools for sequencing, with each pool containing genomes from both parents in the ratio of 1:1, except for the regions harboring QTL of interest (Takagi et al., 2013).

In this study, the QTLseq approach was employed to identify genomic regions and DNA markers associated with resistance to PRSV-W in Nigerian Local accession of *C. moschata*.

## Materials and Methods

### Plant Materials and Phenotyping

A controlled cross between Nigerian Local (resistant accession, maternal) and Butterbush (susceptible Butternut cultivar, paternal) was made in the greenhouse, and a single F_1_ individual was self-pollinated to generate an F_2_ population (*n* = 118). A strain of PRSV-W (provided by Dr. Jane Polston, University of Florida) was maintained on susceptible Yellow Crookneck plants (commercial cultivar, highly susceptible to PRSV) in insect-proof cages (Megaview Science, Talchung, Taiwan). The isolate was confirmed as PRSV-W using enzyme-linked immunosorbent assay (ELISA) (Agdia Inc., Elkhart, IN, United States).

Seeds of each parent (*n* = 12) and the F_2_ population (*n* = 118) were sown in 4-inch pots filled with Proline C/B growing mix (Jolly Gardener, PA, United States) amended with controlled release fertilizer (14:4.2:11.6 NPK). Seeds (*n* = 9) of Yellow Crookneck were also sown to serve as a susceptible check. Inoculation was done at the two true-leaf stages. Fresh inoculum was prepared by grinding leaf tissues of PRSV-W-infected Yellow Crookneck plants in 0.02 M phosphate buffer (pH 7) at 1:5 weight by volume ratio. Cotyledon and first true leaf of the plants were dusted with silicon carbide powder (Thermo Fisher Scientific, MA, United States) and mechanically inoculated with the freshly prepared PRSV-W inoculum. Disease rating (DR) was performed on a scale of 0–3, with 0 representing asymptomatic plants, 1 for plants showing slight yellowing on the leaves, 2 for plants showing moderate yellowing and mottling of the leaves, and 3 for plants with severe leaf yellowing and mottling and stunted plant growth. Disease severity data were recorded 7, 14, 21, and 28 days after inoculation (DAI).

### Whole Genome Re-sequencing and Quantitative Trait Loci Mapping

The DNA was extracted from 10 resistant (DR = 0) and 10 susceptible (DR = 3) F_2_ individuals and the parents using FavorPrep Plant DNA kit (Favorgen Biotech Corp., Ping-Tung, Taiwan) following the manufacturer’s instruction. The resistant and susceptible sequencing libraries were prepared by pooling an equal amount (500 ng) of DNA from individuals constituting the resistant and susceptible bulks, respectively. Whole genome re-sequencing of the parents, resistant, and susceptible bulks was performed on Illumina HiSeq X platform at BGI sequencing center (Shenzhen, Guangdong, China). The resulting paired-end sequencing reads from the parents were mapped into the *C. moschata* (Rifu) genome using BWA-MEM (Li and Durbin, 2009). A consensus reference sequence was generated by replacing *C. moschata* reference alleles with sequence reads from resistant and susceptible parents using SAMtools (Li et al., 2009). Variant calling between the resistant and susceptible bulks was performed following the standard bioinformatic procedures. Briefly, reads from resistant and susceptible bulks were aligned to consensus reference sequence followed by filtering out of duplicate reads using Picard 2.19.1. HaplotypeCaller of GATK4 was used for variant calling (Garrison and Marth, 2012; Van der Auwera et al., 2013). The called variants were then compiled using GVCFs to obtain a raw variant VCF file, which was used as raw input data for QTL analysis in QTLseqr (Mansfeld and Grumet, 2018). In QTLseqr, runQTLseqAnalysis function was implemented to identify significant QTL associated with PRSV-W resistance. Filtering criteria set on QTLseqr to filter out the variants with low confidence were “minimum depth ≥ 50, maximum depth ≤ 500, GQ ≥ 99.” The ΔSNP-index for each SNP position was calculated by determining the difference between SNP index of resistant and susceptible bulks (Takagi et al., 2013). SNP index values across the entire genome of resistant and susceptible bulks were calculated with a sliding window average of 1 Mb. Significant QTL were detected at 95 and 99% confidence intervals (Takagi et al., 2013; Mansfeld and Grumet, 2018).

### Variant’s Characterization, Marker Development, and Validation

Annotation and characterization of SNPs identified within QTL region were performed using SnpEff software (Cingolani et al., 2012). Since *C. moschata* (Rifu) reference genome is not present in SnpEff genomic database, a custom database was built using the reference genome and gff3 annotation files available through Cucurbit Genomics Database prior to the analysis. High-effect SNPs identified by SnpEff were further explored for the marker development and QTL validation. A total of 13 high-effect SNPs were converted into Kompetitive allele-specific PCR (KASP) markers using BatchPrimer3 software (Ali and Al-Koofee, 2019; Supplementary Table 1) and tested for the association with PRSV-W resistance in the F_2_ population (*n* = 118). KASP assays were performed in 10-μl reaction volumes containing 5 μl of 2X low rox master mix, 0.16 μl of each forward primers (10 μm), 0.41 μl of reverse primer, 2 μl of genomic DNA (50 ng/μl), and 2.27 μl of H2O. The PCR amplification process consisted of initial incubation at 94°C for 15 min, touchdown step at 94°C for 20 s, and 61°C for 60 s, with a 0.6°C decrease per cycle for 10 cycles, followed by two-stage PCR step of 26 cycles, 94°C for 20 s and 55°C for 60 s. Fluorescent plate reading and cluster calling were performed using LightCycler^®^ 480 Instrument II (Roche Life Sciences, Penzberg, Germany).

The markers were tested for the association with PRSV-W resistance using the Kruskal–Wallis test (*p* < 0.05), whereas the R/qtl package was used for distance estimation and non-parametric interval mapping using the est.map and scanone functions, respectively (Broman et al., 2003; Broman and Sen, 2009). Likelihood of the odd (LOD) values were determined using 1,000 permutations, and the significance thresholds were viewed at 99, 95, and 90 percentiles. The SNP effect on the predicted amino acid sequence was checked using resources available through the Cucurbit Genomics Database.

## Results

### Phenotypic Data

The onset of leaf yellowing was evident on the susceptible parent (Butterbush) and the susceptible check (Yellow Crookneck) at 14 DAI, whereas leaf distortion and plant stunting were observed at 28 DAI (Supplementary Figure 1). On the contrary, plants of the Nigerian Local accession remained asymptomatic throughout the experiment. At 28 DAI, the F_2_ population was segregated into four distinct phenotypic classes. Individuals with a DR of 0 and 1 were considered resistant, whereas individuals with DR of 2 and 3 were considered susceptible (Supplementary Table 2). Of the total 118 F_2_ individuals evaluated for PRSV-W resistance, 54% were susceptible and 46% were resistant. A chi-square test for the susceptible and resistant classes in the F_2_ population fits a 9:7 ratio (*p* = 0.65; 54 resistant and 64 susceptible) (Supplementary Table 3).

### Quantitative Trait Lociseq Analysis

**FIGURE 1:**
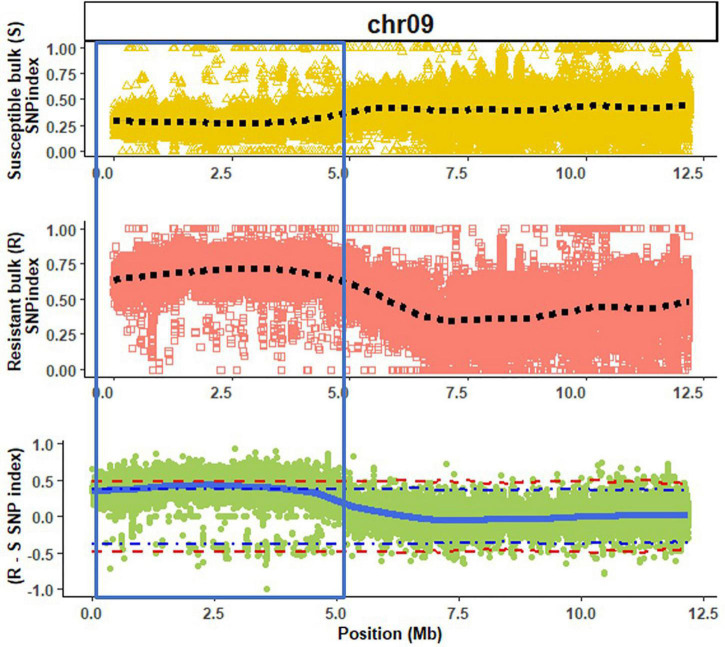
A QTL (*QtlPRSV-C09;* section within blue bar) associated with Papaya ringspot virus W resistance in Nigerian Local (*Cucurbita moschata*) on chromosome 9 (chr09) using Butterbush as the consensus reference genome. The dotted lines (black) represent the smoothed conditional mean for the SNP indexes of susceptible (S) and resistant (R) bulks. The solid blue line represents the tricube ΔSNP index (R SNP index—S SNP index). The blue and red dotted lines in the ΔSNP index plot are the 95 and 99% confidence intervals, respectively.

A high mapping rate (98.07–99.03%) and mapping depth (58.72–79.92) across samples were the indicative of good quality whole genome re-sequencing sequence data (Table 1). The alignment of reads from susceptible and resistant bulks to the Butterbush consensus reference FASTQ file revealed 1,887,568 SNPs. A total of 599,997 SNPs were filtered out by QTLseqr based on the maximum (500) and minimum read depths (50) and genotype quality (GQ =≥ 99). After filtering, 1,287,571 SNPs were utilized in QTLseqr for QTL analysis. The ΔSNP indices across the genome were calculated by subtracting the SNP index of resistant and susceptible bulks at each locus and the values plotted. QTLseq analysis revealed a single QTL (*QtlPRSV-C09*) on chromosome 9 that was significantly (95% confidence interval) associated with PRSV-W resistance in Nigerian Local (Figure 1). *QtlPRSV-C09* extended from 785,532 to 5,093,314 bp and harbored 12,245 SNPs. A similar QTL region was identified using the resistant parent, Nigerian Local, as the consensus reference sequence (Supplementary Figure 2).

**TABLE 1:** Whole genome re-sequencing statistics for resistant and susceptible parents and the corresponding bulks used in the study.

Sample name	Mapping rate (%)	Sequencing depth
Nigerian local	98.07	69.80
Butterbush	98.85	58.72
Susceptible bulk	99.03	79.92
Resistant bulk	98.84	73.81

### Marker-Trait Association

Of the 12,245 SNPs within *QtlPRSV-C09*, 2,004 were missense variants with moderate effect, 3,082 were synonymous variants with low effect, 7,065 were intron variants with modifier effect, and 94 SNPs were variants with high effect. Further examination of the high effect SNPs revealed that 50 were stop-gain, 10 start-loss, 20 splice acceptor, 11 splice donor, and 3 stop loss variants. Only three of the 94 high effect SNPs were located within genes that were previously annotated as putative TIR-NBS-LRR disease-resistant homologs (Supplementary Table 4).

**FIGURE 2:**
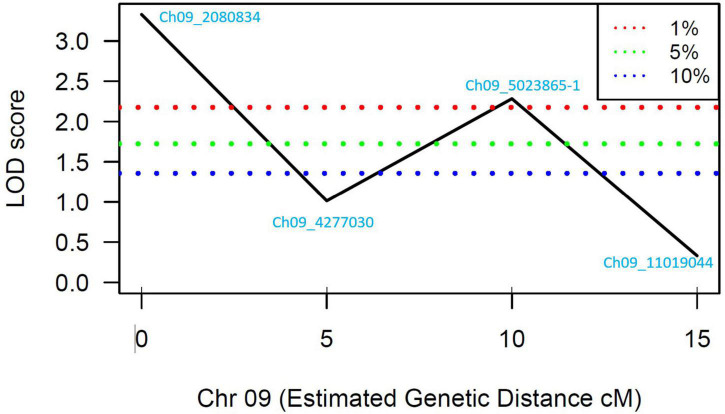
LOD scores for the four SNP markers genotyped in the individuals constituting the resistant and susceptible bulks. The blue, green, and red dotted lines represent 90, 95, and 99% confidence intervals, respectively.

**FIGURE 3:**
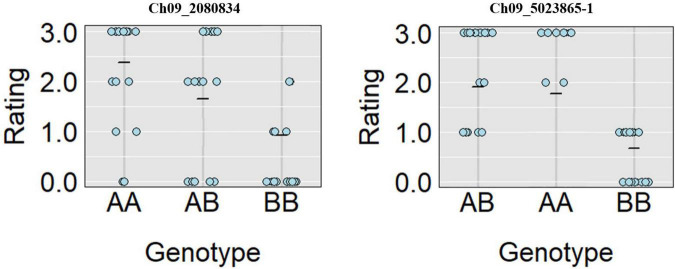
Genotype plots for Ch09_2080834 and Ch09_5023865-1 SNPs showing the corresponding phenotypic distribution in the F_2_ population.

A total of 13 high effect SNPs, which include those identified within putative disease resistance genes, were converted into KASP assays and tested for the association with PRSV-W resistance. A total of four among these were polymorphic between the parents and individuals constituting two bulks and thus were tested in the entire F_2_ population. A number of two SNP markers (Ch09_2080834 and Ch09_5023865-1) within *QtlPRSV-C09* were significantly (*p* < 0.05) associated with PRSV-W resistance in Nigerian Local (Table 2). Non-parametric interval mapping confirmed a significant association of the two markers with PRSV-W resistance (Figure 2). Genotype and phenotype plots of the two SNPs (Ch09_2080834 and Ch09_5023865-1) in the F_2_ population revealed that individuals with the homozygous genotype (BB; SNP from C to T for Ch09_2080834 and SNP from T to C for Ch09_5023865-1) exhibited a higher average disease resistance than those with either the AA (allele C for Ch09_2080834 and allele T for Ch09_5023865-1) or heterozygous genotypes for both markers (Figure 3).

**TABLE 2:** Chromosomal position and Kruskal–Wallis test *p*-values of four SNP markers tested for association with PRSV-W resistance in the F_2_ population between Nigerian Local and Butterbush.

Marker	Genomic position (bp)^a^	Mutation	*p*-value
Ch09_2080834	2,080,834	C/T	0.0004211*
Ch09_4277030	4,277,030	C/A	0.3203
Ch09_5023865-1	5,023,865	T/C	0.003962*
Ch09_11019044	11,019,044	C/T	0.5143

*Significant association of marker with PRSV-W resistance (p < 0.05). ^a^Position of marker in Cucurbita moschata (Rifu) genome.

### Candidate Genes for Papaya Ringspot Virus W Resistance

Further analysis revealed that *QtlPRSV-*C09 contained 70 genes (Supplementary Table 4). The two significant SNPs (Ch09_2080834 and Ch09_5023865-1) were located within putative disease-resistant gene homologs, CmoCh09G004640 and CmoCh09G009540, respectively. CmoCh09G004640 is a clathrin assembly family protein extending from 2,080,680 to 2,083,518 bp on chromosome 9 and shares 49.3% homology with the clathrin assembly family protein of Arabidopsis (*Arabidopsis thaliana*) that features an AP180 N-terminal homology (ANTH) domain. SNP marker Ch09_2080834 [C (susceptible)/T (resistant)] is a stop-gain variant on exon 6 of CmoCh09G004640, which results in a premature stop codon in the resistant parent (Supplementary Figure 3A). On the other hand, homolog CmoCh09G009540 (5,023,313–5,023,867 bp) associated with SNP marker Ch09_5023865-1 is an actin cytoskeleton-regulatory complex gene. SNP Ch09_5023865-1 [T (susceptible)/C (resistant)] is a stop-loss variant located on exon 1 of CmoCh09G009540, which leads to the loss of a stop codon and an elongated amino acid chain in the resistant parent (Supplementary Figure 3B).

## Discussion

PRSV-W is one of the most destructive potyviruses in cucurbit production worldwide (Purcifull, 1984). Genetic resistance to PRSV-W in Nigerian Local (*C. moschata*) is well characterized and widely utilized in breeding (Munger and Provvidenti, 1987; Brown et al., 2003; Behera et al., 2012; Seda-Martinez, 2019). The development and deployment of molecular markers associated with PRSV-W resistance could increase the rate of genetic gain by shortening the length of the selection cycle (Collard et al., 2005). In this study, the QTLseq method was employed to rapidly identify genomic loci associated with resistance to PRSV-W in Nigerian Local. As expected, the phenotypic data confirmed high resistance in Nigerian Local and susceptibility in the susceptible parent (Butterbush) and the cultivar check, Yellow Crookneck. The 9:7 (susceptible/resistant) segregation ratio observed in the F_2_ indicates an oligogenic recessive epistatic mode of inheritance and supports previous inheritance studies in Nigerian Local (McPhail-Medina et al., 2012). On the contrary, Brown et al. (2003) reported that resistance in Nigerian Local is controlled by a single recessive gene (*prv*). The disparities in conclusions among these studies may be due to contrasting thresholds set for resistance and susceptible classes and also the differences in the susceptible parent genetic backgrounds which may contribute to modifying loci (McPhail-Medina et al., 2012).

Although the segregation pattern in the F_2_ was oligogenic, only single QTL (*QtlPRSV-C09*) linked to PRSV-W resistance in Nigerian Local was detected on chromosome 9. This may be due to the small mapping population (*n* = 118) utilized in this study that may have diminished the statistical power for the detection of QTL of minor effect (Vales et al., 2005; Meru and McGregor, 2013). The location of *QtlPRSV-C09* on chromosome 9 is different from the QTL for ZYMV resistance in Nigerian Local on chromosomes 2, 4, 8, and 20 (Shrestha et al., 2021). This observation supports a previous study that demonstrated that the resistance to PRSV-W and ZYMV in Nigerian Local is non-allelic (Brown et al., 2003; Seda-Martinez, 2019).

*QtlPRSV-C09* spanned 4.31 Mb and contained 12,245 SNPs, among which 94 were determined as high-effect SNPs using SnpEff, a tool for delimiting confidence intervals of QTL regions in linkage mapping (;Cingolani et al., 2012). The abundance of SNPs in the non-coding region with no effect was expected because of low sequence conservation and decreased selection pressure in these regions as compared to genic regions (Loewe and Hill, 2010). Among the 13 high-effect SNPs assayed as KASP markers in the F_2_ population, two (Ch09_2080834 and Ch09_5023865-1) were significantly associated with PRSV-W resistance in Nigerian Local. These SNPs, together with those linked to ZYMV resistance in Nigerian Local (Shrestha et al., 2021), can be targeted through MAS to facilitate gene pyramiding for resistance to PRSV and ZYMV in squash. SNP Ch09_2080834 lies within a clathrin assembly family protein (CmoCh09G004640) that features ANTH domain that is associated with clathrin coat assembly and clathrin-dependent endocytosis in Arabidopsis (Mayer et al., 2004; Cheng et al., 2017). Interestingly, three different pattern-recognition receptor kinases, Flagellin sensing 2, PEP receptor 1, and elongation factor-Tu involved in plant defense, are activated *via* clathrin-dependent endocytosis (Mbengue et al., 2016; Ortiz-Morea et al., 2016). On the other hand, SNP Ch09_5023865-1 was within CmoCh09G009540, an actin cytoskeleton-regulatory complex gene reportedly involved in the regulation of leaf senescence, salicylic acid, and pathogen attack in Arabidopsis and barley (*Hordeum vulgare*) (Krupinska et al., 2002; Fischer-Kilbienski et al., 2010). SNPs Ch09_2080834 and Ch09_5023865-1 are non-synonymous variants, which results in stop-gain and stop-loss in CmoCh09G004640 and CmoCh09G009540, respectively, and may result in altered pattern recognition in response to PRSV-W infection in susceptible and resistant parents (Cingolani et al., 2012).

## Conclusion

This study demonstrated QTLseq as a rapid tool to map traits in horticultural crops, particularly for oligogenic traits. Segregation pattern in the F_2_ population indicated that at least two genes are involved in PRSV-W resistance in Nigerian Local. A single QTL (*QtlPRSV-C09*) associated with resistance to PRSV-W resistance was mapped on chromosome 9 of *C. moschata*. The two SNP markers (Ch09_2080834 and Ch09_5023865-1) tightly linked to *QtlPRSV-C09* in Nigerian Local may expedite the development of elite PRSV-W-resistant squash cultivars through MAS.

## Data Availability Statement

Raw fastq data files of resistant and susceptible pools and vcf file associated with PRSV resistance in Nigerian Local X Butterbush F2 are available publicly and can be found in the Figshare data repository. Available at: https://figshare.com/articles/dataset/Sbulk_R1_fq_zip/19241559: Raw reads of F2 individuals (Susceptible pool *n* = 10) derived by crossing *C. moschata* (Nigerian Local X Butterbush) (Read 1). https://figshare.com/articles/dataset/Sbulk_R2_fq_gz_zip/19237527: Raw reads of F2 individuals (Susceptible pool *n* = 10) derived by crossing *C. moschata* (Nigerian Local X Butterbush) (Read 2). https://figshare.com/articles/dataset/Raw_reads_of_Cucurbita_moschata_Butterbush_/19236774?file=34179372: Raw reads of F2 individuals (Resistant pool *n* = 10) derived by crossing *C. moschata* (Nigerian Local X Butterbush) (Read 1). https://figshare.com/articles/dataset/Raw_reads_of_Cucurbita_moschata_Butterbush_/19236774?file=34179501: Raw reads of F2 individuals (Resistant pool *n* = 10) derived by crossing *C. moschata* (Nigerian Local X Butterbush) (Read 2). https://figshare.com/articles/dataset/BB_R_S_raw_snps_and_indels_2_vcf/19236510: SNPs and Indels associated with PRSV resistance in Cucurbita moschata.

## Author Contributions

GM, SS, and YF designed the research. SS, YF, and VM conducted the experiments and analyzed the data. GM and SS wrote the manuscript. All authors read and approved the manuscript.

## Conflict of Interest

The authors declare that the research was conducted in the absence of any commercial or financial relationships that could be construed as a potential conflict of interest.

## Publisher’s Note

All claims expressed in this article are solely those of the authors and do not necessarily represent those of their affiliated organizations, or those of the publisher, the editors and the reviewers. Any product that may be evaluated in this article, or claim that may be made by its manufacturer, is not guaranteed or endorsed by the publisher.

## Supplementary Material

Supplementary Figure 1 The **(A)** susceptible (Butterbush) and **(B)** resistant (Nigerian Local) parents 28 days after mechanical inoculation of PRSV-W virus.

Supplementary Figure 2 A QTL (*QtlPRSV-C09;* region within blue bar) associated with Papaya ringspot virus W resistance in Nigerian Local (*Cucurbita moschata*) on chromosome 9 (chr09) using Nigerian Local as the consensus reference genome. The dotted lines (black) represent the smoothed conditional mean for SNP indexes of susceptible (S) and resistant (R) bulks. The solid blue line represents the tricube ΔSNP index (R SNP index—S SNP index). The blue and red dotted lines in the ΔSNP index plot are the 95 and 99% confidence intervals, respectively.

Supplementary Figure 3 Effect of Ch09_2080834 and Ch09_5023865-1 SNPs on predicted amino acid sequence on CmoCh09G004640 and CmoCh09G009540 homologs, respectively. The highlighted positions indicate the impact of SNP variants in the amino acid sequence where **(A)** SNP Ch09_2080834 results in a stop-gain variant resulting in a premature stop codon stop in the resistant parent and **(B)** SNP Ch09_5023865-1 is a stop-loss variant leading to an elongated polypeptide in the resistant parent.

## References

[B1] Ali S. K. Al-Koofee D. A. (2019). BatchPrimer3: A free web application for allele specific (SBE and allele flanking) primer design for SNPs genotyping in molecular diagnostics: A bioinformatics study. *Gene Rep.* 17:100524. 10.1016/j.genrep.2019.100524

[B2] Behera T. K. Sureja A. K. Islam S. Munshi A. D. Sidhu A. S. (2012). “Minor cucurbits,” in *Genetics, Genomics and Breeding of Cucurbits*, eds Wang Y. Behera T. K. Kole C. (New York, N.Y: CRC Press), 17–60. 10.1201/b11436-3

[B3] Boyhan G. E. Granberry D. M. Kelley W. T. (2009). *Squash: Commercial Vegetable Production. Circular* 527. University of Georgia Extension. Available online at: https://extension.uga.edu/publications/detail.html?number=C527&title=Commercial%20Squash%20Production (accessed October 09, 2021).

[B4] Broman K. W. Sen S. (2009). *A Guide to QTL Mapping with R/qtl* (Vol. 46). New York: Springer.

[B5] Broman K. W. Wu H. Sen Ś Churchill G. A. (2003). R/qtl: QTL mapping in experimental crosses. *Bioinformatics* 19 889–890. 10.1093/bioinformatics/btg112 12724300

[B6] Brown R. N. Bolanos-Herrera A. Myers J. R. Jahn M. M. (2003). Inheritance of resistance to four cucurbit viruses in Cucurbita moschata. *Euphytica* 129 253–258.

[B7] Buermans H. P. J. Den Dunnen J. T. (2014). Next generation sequencing technology: advances and applications. *Biochim. Biophys. Acta* 1842 1932–1941. 10.1016/j.bbadis.2014.06.015 24995601

[B8] Cheng C. Y. Krishnakumar V. Chan A. P. Thibaud-Nissen F. Schobel S. Town C. D. (2017). Araport11: a complete reannotation of the Arabidopsis thaliana reference genome. *Plant J.* 89 789–804. 10.1111/tpj.13415 27862469

[B9] Cingolani P. Platts A. Wang L. L. Coon M. Nguyen T. Wang L. (2012). A program for annotating and predicting the effects of single nucleotide polymorphisms, SnpEff: SNPs in the genome of Drosophila melanogaster strain. *Fly* 6 80–92. 10.4161/fly.19695 22728672 PMC3679285

[B10] Collard B. C. Jahufer M. Z. Z. Brouwer J. B. Pang E. C. K. (2005). An introduction to markers, quantitative trait loci (QTL) mapping and marker-assisted selection for crop improvement: the basic concepts. *Euphytica* 142 169–196. 10.1007/s10681-005-1681-5

[B11] Fischer-Kilbienski I. Miao Y. Roitsch T. Zschiesche W. Humbeck K. Krupinska K. (2010). Nuclear targeted AtS40 modulates senescence associated gene expression in Arabidopsis thaliana during natural development and in darkness. *Plant Mol. Biol.* 73 379–390. 10.1007/s11103-010-9618-3 20238146

[B12] Garrison E. Marth G. (2012). Haplotype-based variant detection from short-read sequencing. *arXiv.* [Preprint]. 10.48550/arXiv.1207.3907

[B13] Kates H. R. (2019). Pumpkins, squashes, and gourds (Cucurbita L.) of North America. *North Am. Crop Wild Relat.* 2 195–224. 10.1007/978-3-319-97121-6_6

[B14] Krupinska K. Haussuhl K. Schafer A. van der Kooij T. A. Leckband G. Lörz H. (2002). A novel nucleus-targeted protein is expressed in barley leaves during senescence and pathogen infection. *Plant Physiol.* 130 1172–1180. 10.1104/pp.008565 12427984 PMC166638

[B15] Li H. Durbin R. (2009). Fast and accurate short read alignment with Burrows–Wheeler transform. *Bioinformatics* 25 1754–1760. 10.1093/bioinformatics/btp324 19451168 PMC2705234

[B16] Li H. Handsaker B. Wysoker A. Fennell T. Ruan J. Homer N. (2009). The sequence alignment/map format and SAMtools. *Bioinformatics* 25 2078–2079. 10.1093/bioinformatics/btp352 19505943 PMC2723002

[B17] Lim T. K. (2012). “Cucurbita moschata,” in *Edible Medicinal And Non-Medicinal Plants*, (Dordrecht: Springer), 266–280. 10.1007/978-94-007-1764-0_41

[B18] Loewe L. Hill W. G. (2010). The population genetics of mutations: good, bad and indifferent. *Philos. Trans. R. Soc. B.* 365 1153–1167. 10.1098/rstb.2009.0317 20308090 PMC2871823

[B19] Mansfeld B. N. Grumet R. (2018). QTLseqr: an R package for bulk segregant analysis with next-generation sequencing. *Plant Genome* 11:180006. 10.3835/plantgenome2018.01.0006 30025013 PMC12810111

[B20] Martinez I. Michael V. N. Fu Y. Q. Shrestha S. Meru G. (2021). DNA Extraction from a Single Seed for Marker-Assisted Selection in Squash. *Am. J. Plant Sci.* 12 1912–1925. 10.4236/ajps.2021.1212132

[B21] Mayer K. Schüller C. Wambutt R. Murphy G. Volckaert G. Pohl T. (1999). Sequence and analysis of chromosome 4 of the plant *Arabidopsis thaliana. Nature* 402, 769–777. 10.1038/47134 10617198

[B22] Mbengue M. Bourdais G. Gervasi F. Beck M. Zhou J. Spallek T. (2016). Clathrin-dependent endocytosis is required for immunity mediated by pattern recognition receptor kinases. *Proc. Natl. Acad. Sci.* 113 11034–11039. 10.1073/pnas.1606004113 27651493 PMC5047200

[B23] McPhail-Medina R. Wessel-Beaver L. Rodrigues J. C. V. (2012). “Inheritance of resistance to Papaya ringspot virus in tropical pumpkin is controlled by at least two genes. In *Cucurbitaceae 2012*,” in *Proceedings of the Xth EUCARPIA Meeting on Genetics and Breeding of Cucurbitaceae, Antalya, Turkey*, (Adana: University of Cukurova), 697–701.

[B24] Meru G. McGregor C. (2013). Genetic mapping of seed traits correlated with seed oil percentage in watermelon. *HortScience* 48 955–959. 10.21273/hortsci.48.8.955

[B25] Meru G. McDowell D. Waters V. Seibel A. Davis J. McGregor C. (2013). A non-destructive genotyping system from a single seed for marker-assisted selection in watermelon. *Gene. Mol. Res.* 12 702–709. 10.4238/2013.March.11.18 23546952

[B26] Munger H. M. Provvidenti R. (1987). Inheritance of resistance to zucchini yellow mosaic virus in Cucurbita moschata. *Cucurbit Genet. Coop. Rep.* 10 80–81. 10.1093/jhered/esr006 21493595

[B27] Nigam D. LaTourrette K. Souza P. F. Garcia-Ruiz H. (2019). Genome-wide variation in potyviruses. *Front. Plant Sci.* 10:1439. 10.3389/fpls.2019.01439 31798606 PMC6863122

[B28] Ortiz-Morea F. A Savatin D. V Dejonghe W Kumar R Luo Y Adamowski M (2016). Danger-associated peptide signaling in Arabidopsis requires clathrin. *Proc. Natl. Acad. Sci.* 39 11028–11033. 10.1073/pnas.1605588113 27651494 PMC5047203

[B29] Pachner M. Paris H. S. Lelley T. (2011). Genes for resistance to zucchini yellow mosaic in tropical pumpkin. *J. Heredity* 102 330–335. 10.1093/jhered/esr006 21493595

[B30] Paris H. S. (1996). Summer squash: history, diversity, and distribution. *HortTechnology* 6 6–13. 10.21273/horttech.6.1.6

[B31] Paris H. S. (2008). “Summer squash,” in *Vegetables I. Handbook of Plant Breeding*, Vol. 3, eds Prohens J. Nuez F. (New York, NY: Springer), 351–375.

[B32] Purcifull D. E. (1984). Papaya ringspot virus. CMI/AAB Descriptions of plant viruses. *Descr. Plant Viruses* 209:8.

[B33] Ramos A. Fu Y. Michael V. Meru G. (2020). QTL-seq for identification of loci associated with resistance to Phytophthora crown rot in squash. *Sci. Rep.* 10 1–8. 10.1038/s41598-020-62228-z 32210312 PMC7093484

[B34] Rubio L. Galipienso L. Ferriol I. (2020). Detection of plant viruses and disease management: relevance of genetic diversity and evolution. *Front. Plant Sci.* 11:1092. 10.3389/fpls.2020.01092 32765569 PMC7380168

[B35] Sáez C. Martínez C. Montero-Pau J. Esteras C. Sifres A. Blanca J. (2020). A major QTL located in chromosome 8 of Cucurbita moschata is responsible for resistance to Tomato leaf curl New Delhi virus. *Front. Plant Sci.* 11:207. 10.3389/fpls.2020.00207 32265946 PMC7100279

[B36] Seda-Martinez W. (2019). *The Effect of Two Potyviruses on Development and Yield in Tropical Pumpkin and the Inheritance of Resistance to Papaya Ringspot virus.* Ph.D thesis. Mayaguez PR: University of Puerto Rico.

[B37] Seda-Martínez W. Wessel-Beaver L. Linares-Ramírez A. Rodrigues J. C. V. (2021). Virus Quantification, Flowering, Yield, and Fruit Quality in Tropical Pumpkin (Cucurbita moschata Duchesne) Genotypes Susceptible or Resistant to Two Potyviruses. *HortScience* 56 193–203. 10.21273/hortsci15525-20

[B38] Shrestha S. Michael V. N. E. Fu Y. Meru G. (2021). Genetic Loci Associated with Resistance to Zucchini Yellow Mosaic Virus in Squash. *Plants* 10:1935. 10.3390/plants10091935 34579467 PMC8465829

[B39] Stevenson D. G. Eller F. J. Wang L. Jane J. L. Wang T. Inglett G. E. (2007). Oil and tocopherol content and composition of pumpkin seed oil in 12 cultivars. *J. Agricult. Food Chem.* 55 4005–4013. 10.1021/jf0706979 17439238

[B40] Sun H. Wu S. Zhang G. Jiao C. Guo S. Ren Y. (2017). Karyotype stability and unbiased fractionation in the paleo-allotetraploid Cucurbita genomes. *Mol. Plant* 10 1293–1306. 10.1016/j.molp.2017.09.003 28917590

[B41] Takagi H. Abe A. Yoshida K. Kosugi S. Natsume S. Mitsuoka C. (2013). QTL-seq: rapid mapping of quantitative trait loci in rice by whole genome resequencing of DNA from two bulked populations. *The Plant Journal* 74 174–183. 10.1111/tpj.12105 23289725

[B42] USDA-National Agricultural Statistics Service [NASS] (2021). *2020 Census of Agriculture.* https://quickstats.nass.usda.gov/. (accessed October 9, 2021).

[B43] Vales M. I. Schön C. C. Capettini F. Chen X. M. Corey A. E. Mather D. E. (2005). Effect of population size on the estimation of QTL: a test using resistance to barley stripe rust.”. *Theor. Appl. Gen.* 7 1260–1270. 10.1007/s00122-005-0043-y 16179997

[B44] Van der Auwera G. A. Carneiro M. O. Hartl C. Poplin R. Del Angel G. Levy-Moonshine A. (2013). From FastQ data to high-confidence variant calls: the genome analysis toolkit best practices pipeline. *Curr. Prot. Bioinform.* 43 11–10. 10.1002/0471250953.bi1110s43 25431634 PMC4243306

[B45] Vinayashree S. Vasu P. (2021). Biochemical, nutritional and functional properties of protein isolate and fractions from pumpkin (Cucurbita moschata var. Kashi Harit) seeds. *Food Chem.* 340:128177. 10.1016/j.foodchem.2020.128177 33002826

[B46] Vogel G. LaPlant K. E. Mazourek M. Gore M. A. Smart C. D. (2021). A combined BSA-Seq and linkage mapping approach identifies genomic regions associated with Phytophthora root and crown rot resistance in squash. *Theor. Appl. Genet*. 134 1015–1031. 10.1007/s00122-020-03747-1 33388885

[B47] Whitaker T. W. Robinson R. W. (1986). “Squash breeding,” in *Breeding Vegetable Crops Westport*, ed. Basset M. J. (Westport, CT: Avi Publishing Company).

[B48] Yadav M. Jain S. Tomar R. Prasad G. B. K. S. Yadav H. (2010). Medicinal and biological potential of pumpkin: an updated review. *Nutr. Res. Rev.* 23 184–190. 10.1017/S0954422410000107 21110905

