# Supplementary figures and images for: Whole Genome Re-sequencing and Bulk Segregant Analysis Reveals Chromosomal Location for Papaya Ringspot Virus W Resistance in Squash
Source: Front Plant Sci. 2022 May 19;13:848631. doi: 10.3389/fpls.2022.848631 (PMC9161299; doi:10.3389/fpls.2022.848631)

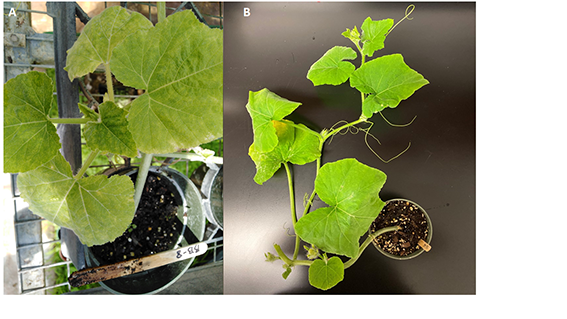

Supplement: Supplementary Figure 1 — The (A) susceptible (Butterbush) and (B) resistant (Nigerian Local) parents 28 days after mechanical inoculation of PRSV-W virus. [file Image_1.JPEG]

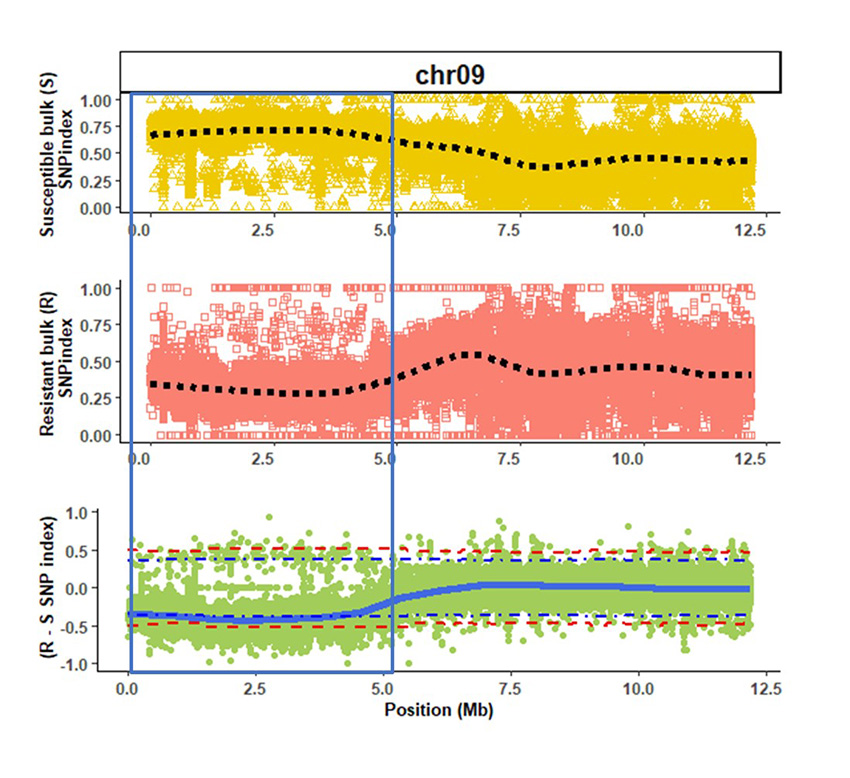

Supplement: Supplementary Figure 2 — A QTL (QtlPRSV-C09; region within blue bar) associated with Papaya ringspot virus W resistance in Nigerian Local (Cucurbita moschata) on chromosome 9 (chr09) using Nigerian Local as the consensus reference genome. The dotted lines (black) represent the smoothed conditional mean for SNP indexes of susceptible (S) and resistant (R) bulks. The solid blue line represents the tricube ΔSNP index (R SNP index—S SNP index). The blue and red dotted lines in the ΔSNP index plot are the 95 and 99% confidence intervals, respectively. [file Image_2.JPEG]

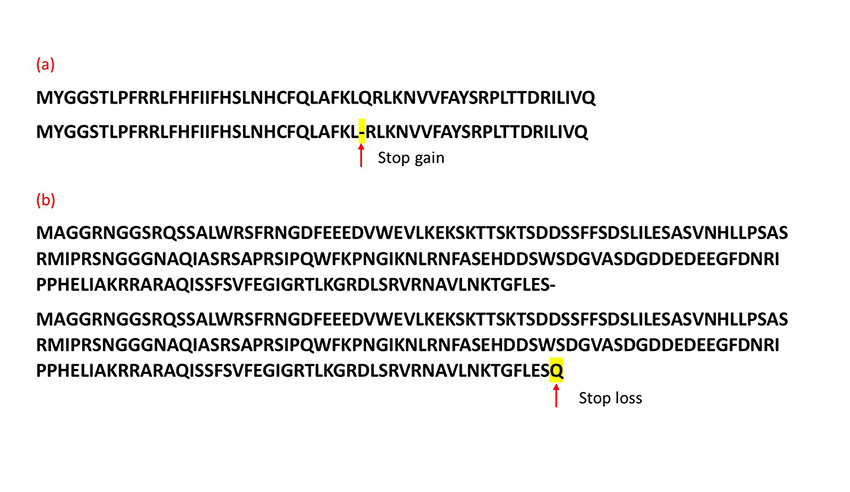

Supplement: Supplementary Figure 3 — Effect of Ch09_2080834 and Ch09_5023865-1 SNPs on predicted amino acid sequence on CmoCh09G004640 and CmoCh09G009540 homologs, respectively. The highlighted positions indicate the impact of SNP variants in the amino acid sequence where (A) SNP Ch09_2080834 results in a stop-gain variant resulting in a premature stop codon stop in the resistant parent and (B) SNP Ch09_5023865-1 is a stop-loss variant leading to an elongated polypeptide in the resistant parent. [file Image_3.JPEG]
